# Cancer immunotherapy beyond immune checkpoint inhibitors

**DOI:** 10.1186/s13045-017-0552-6

**Published:** 2018-01-12

**Authors:** Julian A. Marin-Acevedo, Aixa E. Soyano, Bhagirathbhai Dholaria, Keith L. Knutson, Yanyan Lou

**Affiliations:** 1 Department of Internal Medicine, Mayo Clinic, Jacksonville, FL USA; 2 Department of Hematology and Oncology, Mayo Clinic, 4500 San Pablo Road, Jacksonville, FL 32224 USA; 3 Current address: Department of Blood and Marrow Transplant and Cellular Immunotherapy, Moffitt Cancer Center, Tampa, FL USA; 4 Department of Immunology, Mayo Clinic, Jacksonville, FL USA

**Keywords:** Immunotherapy, Tumor-directed monoclonal antibodies, Antibody drug conjugates, Chimeric antigen receptor therapy, Oncolytic viruses, Tumor vaccines, Viral gene therapy

## Abstract

Malignant cells have the capacity to rapidly grow exponentially and spread in part by suppressing, evading, and exploiting the host immune system. Immunotherapy is a form of oncologic treatment directed towards enhancing the host immune system against cancer. In recent years, manipulation of immune checkpoints or pathways has emerged as an important and effective form of immunotherapy. Agents that target cytotoxic T lymphocyte-associated molecule-4 (CTLA-4), programmed cell death receptor-1 (PD-1), and programmed cell death ligand-1 (PD-L1) are the most widely studied and recognized. Immunotherapy, however, extends beyond immune checkpoint therapy by using new molecules such as chimeric monoclonal antibodies and antibody drug conjugates that target malignant cells and promote their destruction. Genetically modified T cells expressing chimeric antigen receptors are able to recognize specific antigens on cancer cells and subsequently activate the immune system. Native or genetically modified viruses with oncolytic activity are of great interest as, besides destroying malignant cells, they can increase anti-tumor activity in response to the release of new antigens and danger signals as a result of infection and tumor cell lysis. Vaccines are also being explored, either in the form of autologous or allogenic tumor peptide antigens, genetically modified dendritic cells that express tumor peptides, or even in the use of RNA, DNA, bacteria, or virus as vectors of specific tumor markers. Most of these agents are yet under development, but they promise to be important options to boost the host immune system to control and eliminate malignancy. In this review, we have provided detailed discussion of different forms of immunotherapy agents other than checkpoint-modifying drugs. The specific focus of this manuscript is to include first-in-human phase I and phase I/II clinical trials intended to allow the identification of those drugs that most likely will continue to develop and possibly join the immunotherapeutic arsenal in a near future.

## Background

**Fig. 1:**
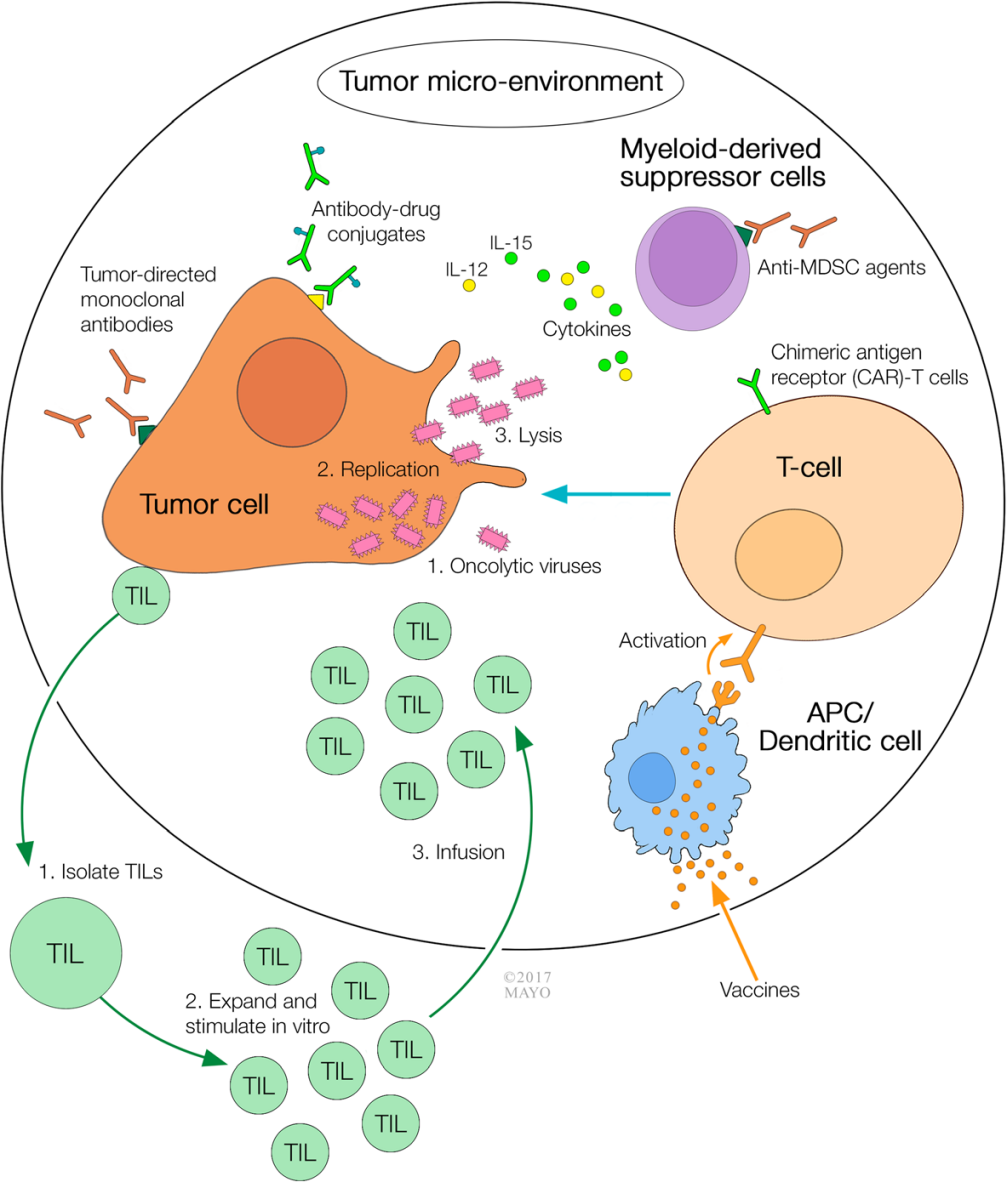
Multi-modality cancer immunotherapy approaches

Immunotherapy consists in harnessing the body’s own immune system to generate an anti-tumor response, which is often sustained after treatment has finished, suggesting a role of modulation and modification of the immune system [1, 2]. The most commonly used strategy is the modulation of immune checkpoints, particularly the cytotoxic T lymphocyte-associated molecule-4 (CTLA-4), programmed cell death receptor-1 (PD-1), or programmed cell death ligand-1 (PD-L1), although new inhibitory (e.g., lymphocyte activation gene/LAG-3, T cell immunoglobulin/TIM-3, V-domain Ig suppressor of T cell activation/VISTA) and stimulatory (e.g., inducible co-stimulator/ICOS, OX40, 4-1BB) pathways have also emerged as targets [3]. Unfortunately, some limitations with immune checkpoint therapy are of concern including a response heterogeneity where some patients achieve a complete response (CR) but others never do. Furthermore, there can be tumor relapse due to alternative immune escape mechanisms, and there is lack of optimal biomarkers to predict response and toxicity. Other major issues have been the emergence of new adverse events and autoimmune-like reactions, and the cost associated with this therapy. Thus, approaches that differ from manipulating immune checkpoints, as alternatives, are under investigation and are the focus of this review. Specifically, we discuss current molecules under phase I and I/II clinical investigation in the area of conjugated monoclonal antibodies (mAbs), chimeric antigen receptor (CAR) T cells, oncolytic viruses, vaccines, and other immune-based approaches that are under investigation. Agents in more advanced investigational stages (e.g., phase III) have not been included. Figure 1 summarizes the different strategies that will be discussed, and a summary of these agents is found in Table 1.

**Table 1:** Summary of non-immune checkpoint blockade agents

Category	Drug	Target	Trial	Phase	Type of tumor	Clinical efficacy	Safety	Comments
Tumor-directed monoclonal antibodies								
	Ensituximab	MUC5A	NCT01040000	I/II	CRC and pancreatic	OS improved from 5.0 to 6.8 months, 21/56 pts survived > 12 months	< 2% of patients with grade 3 toxicities and no grade 4 adverse events	Trial completed
	CEA CD3 TCB (RG7802, RO6958688)	CEA and CD3	NCT02324257	I	CEA(+) solid tumors	5% PR	16% patients developed grade 3 or more adverse events	In conjunction with obinutuzumab
			NCT02650713	I	CEA(+) solid tumors	20% PR		In conjunction with atezolizumab
	Blinatumomab	CD19 and CD3	NCT01741792	II	DLBCL	19% CR, PFS up to 20 months	Grade 3 neurologic events (9% encephalopathy, 9% aphasia)	Trial completed
	BAY2010112 (AMG212, MT112)	PSMA and CD3	NCT01723475	I	Castration-resistant prostate cancer	Not reported	–	Study is ongoing but not recruiting
	MOR209/ES414	PSMA and CD3	NCT02262910	I				–
	AFM13	CD30 and CD16A	NCT01221571	I	CD30+ HL	3/26 PR, 13/26 SD, overall DCR 61.5%	Mild to moderate adverse events ranging from fever to infusion reactions	Trial completed
			NCT02321592	II	CD30+ HL	–	–	–
Antibody drug conjugates								
	ABBV-339	c-Met	NCT02099058	I	NSCLC	19% PR	All-grade adverse events in > 10% of patients	Used in conjunction with erlotinib
	Glembatumumab vedotin (GV, CDX-011)	gpNMB	NCT02302339	II	Melanoma	1/62 CR, 6/62 PR, and 33/62 SD	Alopecia, neuropathy, rash, fatigue and neutropenia	–
	Losatuxizumab vedotin (ABBV-221)	EGFR	NCT02365662	I	EGFR-dependent tumors	38% SD and 1 patient had unconfirmed PR	Infusion reactions and fatigue	–
	Mirvetuximab soravtansine (IMGN853)	Folate receptor alpha (FRα)	NCT01609556 (FORWARD I)	I	Ovarian cancer	ORR 46%	1/37 CR and 16/37 PR	Monotherapy
			NCT02606305 (FORWARD II)	I	Ovarian cancer	–	Most adverse events were grade 2 or less	Used in combination with bevacizumab, carboplatin, liposomal doxorubicin, or pembrolizumab
	Enfortumab vedotin (ASG-22CE; ASG-22ME)	Nectin-4	NCT02091999	I	Urothelial tumors	ORR 40%, CR 3/68, median duration of response was 18 weeks, and median PFS 17 weeks	85% developed adverse events, but most were grade 2 or less	–
	Sacituzumab govitecan (IMMU-132)	Trop-2	NCT01631552	I/II	Epithelial cell tumors	30% ORR, 2/69 CR, 19/69 PR, median OS 16.6 months for triple negative breast cancer	Neutropenia, diarrhea, febrile neutropenia	–
						ORR 19%, median		
						PFS 5.2 months, and a median OS of 9.5 months in NSCLC		
	Inotuzumab ozogamicin (InO/CMC-544)	CD22	NCT01055496	I	CD22+ NHL	ORR 53%	85% thrombocytopenia, 69% of neutropenia	Used in conjunction with rituximab, gemcitabine, dexamethasone, and cisplatin Trial completed
	Labetuzumab govitecan (IMMU-130)	CEACAM	NCT01605318	II	CRC	1/86 PR, 42/86 had SD, OS was 6.9 months, and PFS was 3.6 months	16% Neutropenia), 9% anemia, and 7% diarrhea	–
	Lorvotuzumab mertansine (IMGN901)	CD56	NCT01237678	I/II	SCLC	94 patients (combination ADC with chemotherapy) and 47 patients (no ADC) achieved a PFS of 6.2 and 6.7 months, respectively Median OS was 10 months in both cohorts	29% Peripheral neuropathy , 21/94 patients had a treatment-emergent adverse event leading to death	Used in combination with carboplatin and etoposide
	Rovalpituzumab tesirine (Rova-T)	DLL3	NCT02674568	II	SCLC	18% ORR, and 54% SD	38% developed serious adverse events (pleural and pericardial effusions)	Ongoing
			NCT03026166	I	SCLC	–	–	Used in combination with nivolumab or nivolumab and ipilimumab
	ADCT-301	CD25	NCT02432235	I	HL and NHL	1/18 CR, 1/18	Rash, mucositis, enteritis, and elevated CPK	-
						PR, 6/18 SD		
			NCT02588092	I	AML and ALL	–	–	–
	TAK-264 (MLN0264)	GCC	NCT02202785	II	Pancreatic adenocarcinoma	3% ORR	All patients developed at least 1 adverse event	Trial was terminated
	MEDI-4276	HER2	NCT02576548	I/II	HER2+ solid tumors	–	–	–
	XMT-1522	HER2	NCT02952729	I	HER2+ breast cancer	–	–	–
	ARX-788	HER2	NCT02512237	I	HER2+ c1ancers	–	–	–
	DS-8201a	HER2	NCT02564900	I	Solid tumors	–	–	–
	SDY985	HER2	NCT02277717	I	Solid tumors	–	–	–
	ADCT-502	HER2	NCT03125200	I	HER2+ solid tumors	–	–	–
	Glembatumumab vedotin (CDX-011, CR-11-vc-MMAE)	GPNMB	NCT02302339	II	Melanoma	1/62 CR, 6 PR, 33 SD, and median OS was 9.8 months	Alopecia, neutropenia, and rash	–
			NCT01156753	II	Advanced GPNMB-expressing breast cancer	ORR 6%	Rash and pruritus	Trial completed
	SAR566658	CA-6	NCT01156870	II	Solid tumors expressing CA6	1/114 CR, 8 PR, and 39% SD	Mild toxicities: fatigue, neuropathy, neutropenia	Trial completed
	SGN-LIV1A	LIV-1	NCT01969643	I	Breast cancer	ORR 11% and SD or better achieved in 63% of patients	No dose-limiting toxicities	Used in combination with trastuzumab
	PF-06647020	Tyrosine kinase 7	NCT02222922	I	Advanced solid tumors	1/76 CR, 5 PR, 12 SD, and 4 PD	Most toxicities were mild: nausea, alopecia, neutropenia	–
	PF-6647263	Ephrin-A4	NCT02078752	I	Advanced solid tumors	5/48 PR	Dose-limiting toxicities were observed in 6 patients	Trial completed
	SAR428926	LAMP-1	NCT02575781	I	Solid tumors	–	–	–
	PCA062	P-cadherin 3	NCT02375958	I	Triple negative breast cancer, head and neck cancer, esophageal cancer	–	–	–
	U3-1402	HER3	NCT02980341	I/II	HER3+ metastatic breast Cancer	–	–	–
	HuMax-Axl	Axl	NCT02988817		Ovarian, cervical, endometrial, NSCLC, thyroid cancer, and melanoma	–	–	–
	MEDI3726	PMSA	NCT02991911	I	Metastatic castration-resistant prostate cancer	–	–	Used in combination with enzalutamide
CAR T cells								
	T4 immunotherapy	ErbB dimers, IL4	NCT01818323	I	HNSCC	DCR 44%	All grade 2 (or less) adverse events	Intratumoral T-4 therapy
	CART-19	CD19	NCT01044069	I	B-ALL	CR rates were 95 and 77% on patients with < 5% of blasts in the bone marrow and those with > 5%, respectively	CRS and neurotoxicity	–
			NCT02348216	I/II	NHL	ORR 82% and CR 39% after 8 months	31% Febrile neutropenia, 24% thrombocytopenia, 21% encephalopathy, and 13% CRS	Some patients received steroids and others tocilizumab
			NCT01865617	I/II	ALL, NHL, and CLL	31/33 ALL patients achieved CR, 6/12 CLL with CR, 84% ORR in NHL	16% CRS and 31% neurotoxicity	All CLL pts had received prior ibrutinib
	Anti-GPC3	GPC3	NCT02395250	I	GPC3+ HCC	1/13 with PR	No dose-limiting toxicities	–
	CART-133	CD133	NCT02541370	I	HCC, pancreas, CRC, cholangiocarcinoma	21/23 PFS ranging from 8 to 22 weeks	Hyperbilirubinemia and CRS	–
	bb2121	BCMA	NCT02658929	I	MM	6/11 ORR	Only grade 1–2 CRS	ORR seen in patients who received higher doses of T cells
	Anti-kappa light chain	Kappa light chains	NCT00881920	I	Kappa (+) CLL, NHL, and MM	2/9 CR, 1/9 PR. 4/7 patients with MM had SD	None	–
	Anti-CD30	CD30	NCT01316146	I	HL and NHL	2 patients with HL and 1 w/ ALCL achieved CR, 3 patients with HL achieved SD	None	–
			NCT02690545	Ib/II	CD30+ HL and NHL	–	–	–
	Anti-IL13	IL13Rα2	NCT02208362	I	Glioblastoma	CR in 1 patient	None	–
TCR gene-modified T cell therapy								
	NY-ESO-1c259t	NY-ESO-1 and HLA-A2	NCT01343043	I/II	Sarcoma	ORR 50%, 1 case of CR	96% Leukopenia, 79% anemia, 79% thrombocytopenia, 1 fatal bone marrow failure, and 11/34 cases with CRS	–
	Anti-E6	E6	NCT02280811	I/II	HPV 16+ carcinomas (e.g., cervical, anal, pharyngeal)	2/12 PR	No dose-limiting toxicity	Study completed
	Anti-MAGE A10	MAGE-A10	NCT02989064	I	Urothelial cancer, HNSCC, or melanoma	–	–	–
Tumor-infiltrating cell therapy								
	TIL	Varies depending on tumor	NCT01319565	II	Melanoma	CR24%	13/48 patients who received TBI developed thrombotic microangiopathy not seen in patients with no TBI	In combination with TBI
	MIL	Varies depending on tumor	NCT00566098	I/II	MM	27% CR, 27% PR, 23% SD, and 14% PD	Not mentioned	Study completed
Oncolytic viruses								
	Coxsackievirus A21 (CVA21–CAVATAK)	ICAM-1-expressing tumors	NCT02565992	Ib	Melanoma, NSCLC, bladder, and prostate cancer	ORR 73% and DCR 91% in melanoma	No dose-limiting toxicities	In combination with pembrolizumab
			NCT02043665					
			NCT02307149	I	Melanoma	ORR 38%	Minimal toxicity	In conjunction with ipilimumab
						DCR 88%		
	Pelareorep (Reolysin)	Different tumors	NCT00984464	II	Melanoma	ORR 21%, 1-year survival 43%, DCR85%	Fever was the most common toxicity	In combination with carboplatin and paclitaxel. Study was completed
			NCT01656538	II	Breast cancer	Median OS was 17.4 months for patients with both agents and 10.4 months for patients with paclitaxel alone	Fatigue, nausea, vomiting, diarrhea	In combination with paclitaxel, or paclitaxel alone
	DNX-2401	Glioblastoma	NCT01956734	I	Glioblastoma	1 patient was alive 30 months into treatment and 2 after 23 months	Related to temozolamide or underlying disease	In combination with temozolamide
	Enadenotucirev (EnAd)	Tumors of epithelial origin	NCT02636036	I	Epithelial tumors	–	–	In combination with nivolumab
Vaccines								
	HS-110 (Viagenpumatucel-L)	Lung adenocarcinoma cells	NCT02439450	I/IIb	Lung adenocarcinoma	ORR 50%	Injection site reactions, maculopapular rash	In combination with nivolumab
	gp96	Gastric cancer cells	NCT02317471	II	Gastric cancer	2-year OS was 81.9% in the vaccination arm vs. 67.9% with chemotherapy-alone arm	No clinically significant adverse events	In combination with oxaliplatin
	GM.CD40L	Lung adenocarcinoma cells	NCT02466568	I/II	Lung adenocarcinoma	Median OS was 9.4 months	No dose-limiting toxicities	Some patients had added CCL21 to GM.CD40L
	RNA-lipoplex (RNA(LIP))	Melanoma antigens	NCT02410733	I/II	Melanoma	–	No dose-limiting toxicities	–
	VXM01	VEGFR-2	EudraCT 2011-000222-29	I	Pancreatic cancer	OS was 9.3 months vs 8.4 months (placebo)	Lymphopenia and increased diarrhea	Oral vaccine
	INO-5150	Prostate cancer antigens	NCT02514213	I	Prostate cancer	10% PD	No dose-limiting toxicities	Used with or without IL-12. Study is not recruiting patients
	INVAC-1	Human telomerase	NCT02301754	I	Solid tumors	12/20 SD	Asthenia and local reaction at injection site	–
	pTVG-HP	PAP	NCT01341652	II	Prostate cancer	–	–	Ongoing
	ADXS11-001	E7 antigen	NCT02164461	I	Cervical cancer	–	Only 1 > grade 2 adverse event (hypotension)	Ongoing
	AdMA3	MAGE-A3	NCT02285816	I	Solid tumors	Evidence of induction of pro-inflammatory genes and subsequent anti-tumor activity	4/41 patient developed dose-limiting toxicities (hypoxia, dyspnea, vomiting, headache)	Used in conjunction with an oncolytic virus (MG1MA3)
	AdHER2ECTM	Her2	NCT01730118	I	Her2(+) tumors	1/27 CR, 1 PR, and 5 SD	Local injection site reactions	–
	CMB305	NY-ESO-1	NCT02387125	I	NY-ESO-1-expressing solid tumors	Increase of anti-NY-ESO-1 T cells in 65% of patients and anti-NY-ESO-1 antibodies in 68% of patients	–	–
	MVA	Brachyury	NCT02179515	I	Advanced solid malignancies	82% of patients developed brachyury-specific immune responses	No dose-limiting toxicities were observed	Trial completed
	BPX101	Human prostate-specific membrane antigen	NCT00868595	I	Prostate cancer	1/18 with CR and 2/18 PR	No dose-limiting toxicities	Trial completed
	WT-1	WT-1-expressing tumors	UMIN000005248	II	Pancreatic adenocarcinoma	Increased OS from 21.5 (gemcitabine alone) to 34.2 (gemcitabine with WT-1 vaccine)	–	Used in conjunction with gemcitabine
	WT4869	WT-1-expressing tumors	JapicCTI-101374	I/II	MDS	ORR 18.2%, median OS 64.71 weeks	30.8% Neutropenia, 7.7% febrile neutropenia, and 7.7% elevated CPK	Trial completed
	Galinpepimut-S	WT-1-expressing tumors	NCT01265433	II	Pleural mesothelioma	PFS 45%, median OS 22.8 months	Mild and not clinically significant	Trial completed
	DPX-Survivac	Survivin-expressing tumors	NCT01416038	Ib	Ovarian, fallopian, and peritoneal cancer	Sustained immune responses of varying magnitude and duration	Skin ulceration	Trial completed
	AE37	Her2	NCT00524277	II	Her2(+) breast cancer	Disease-free survival improved from 51% (GM-CSF alone) to 89% (AE37+ GM-CSF)	Vaccines is safe and well tolerated	Used with GM-CSF
	Multi-HLA binding peptides	HSP70 and GPC3	UMIN000020440	I	Solid tumors	Decreased tumor-marker expression in 6/12 patients and disease control in 5/12 patients	No severe toxicities	–
	URLC10-CDCA1-KOC1	URLC10, CDCA1, KOC1	UMIN000003557	II	Esophageal squamous cell carcinoma	No significant difference of relapse-free survival compared to control group, but there was good immunological response	–	–
	Poly-ICLC	TLR-3	NCT01984892	I/II	Solid tumors including melanoma, breast, and HNSCC	1/8 SD for 41 weeks; the remainder of patients showed PD	Mild and limited to the site of application	Study was terminated
	BO-112	MDA-5 and NOXA	NCT02828098	I	Melanoma and breast cancer	–	1 case of reversible thrombocytopenia	–
	IVAC MUTANOME	Personal tumor neoantigens	NCT02035956	I	Melanoma	8/13 patients remained recurrence-free for the entire follow-up period (12–23 months)	No major adverse events	Ongoing
	Immunogenic personal neoantigen vaccine	Personal tumor neoantigens	NCT01970358	I	Melanoma	4/6 patients that had no recurrence of the disease at 25 months after vaccination	Mild flu-like symptoms, injection site reactions, rash, and fatigue	–
Targeting MDSCs								
	DS-8273a	TRAIL-R2 (DR-5)	NCT02076451	I	Solid tumors	–	No dose-limiting toxicities	–
Cytokine gene therapy								
	Ad-RTS-hIL-12	IL-12	NCT02026271	I	Glioblastoma	Median OS 12.5 months	Flu-like illness, grade 3 CRS, transaminitis	Used in with Veledimex
	NKTR-214	IL-2	NCT02983045	I/II	Solid malignancies	1 patient had unconfirmed CR	No dose-limiting toxicities	Used in conjunction with nivolumab
			NCT02869295	I/II	Solid malignancies	23% achieved tumor size reduction ranging from 10–30%	No dose-limiting toxicities	–
Agents targeting tumor microenvironment								
	BMS-986205	IDO	NCT02658890	I	Solid tumors	–	Hepatitis, rash	Used in conjunction with nivolumab
	Indoximod	IDO	NCT02073123	II	Melanoma	ORR 52%	No significant toxicities	Used in conjunction with ipilimumab, nivolumab, or pembrolizumab
			NCT02077881	II	Pancreatic	ORR 37%	One case of colitis	Used with both gemcitabine and nab-paclitaxel
			NCT01560923	II	Prostate	Median PFS increased from 4.1 to 10.3 months	No significant adverse events	–
	Epacadostat	IDO	NCT02327078 NCT02178722	I/II	Solid and hematologic malignancies	ORR of 75% (melanoma) and 4% (CRC)	No dose-limiting toxicities	–
	MEDI9197	TLR7/8	NCT02556463	I	Solid malignancies	–	Mild adverse events only	In combination with durvalumab and radiation therapy
	PG545 (pixatimod, pINN)	TLR9/IL-12	NCT02042781	I	Solid malignancies	SD for 24 weeks, DCR of 38%	Dose-limiting toxicities in 3/23 patients	–
	Poly-ICLC	TLR3	NCT00553683	I	HCC	PFS 66% at 6 months, 28% at 24 months	Most grade I–II adverse events	In combination with radiation therapy
						OS 69% at 1 year, 38% at 2 years		
	CB-1158	Arginase	NCT02903914	I	Solid malignancies	–	No dose-limiting toxicities	In conjunction with nivolumab
Oncolytic peptides								
	LTX-315	Tumor mitochondrial membranes	NCT01986426	I	Melanoma and breast cancer	2/28 CR, 5 patients had a decreased of > 50% of the tumor size, and 8 patients achieved SD	Most common adverse events were mild local erythema, flushing, pruritus, and transient hypotension	In combination with ipilimumab or pembrolizumab

*Abbreviations*: *ALL* acute lymphocytic leukemia, *ALCL* anaplastic large cell lymphoma, *AML* acute myeloid leukemia, *B-ALL* B cell acute lymphocytic leukemia, *CAR* chimeric antigen receptor, *BCMA* B cell maturation antigen, *CEACAM* CEA cell adhesion molecule, *CLL* chronic lymphocytic leukemia, CPK creatine phosphokinase, *CR* complete response; *CRC* colorectal cancer, *DLBCL* diffuse large B cell lymphoma, *CRS* cytokine release syndrome, *DCR* disease control rate, *DLL3* delta-like protein 3, *GCC* guanylyl cyclase C, *GPC3* glypican-3, *gPNMB* glycoprotein non-metastatic B, *HCC* hepatocellular carcinoma, *HD* Hodgkin’s disease, *HNSCC* head and neck squamous cell carcinoma, *IDO* indoleamine 2,3-dioxygenase, *MDS* myelodysplastic syndrome, *MDSCs* myeloid-derived suppressor cells, *MIL* marrow-infiltrating lymphocyte, *MM* multiple myeloma, *NHL* non-Hodgkin’s lymphoma, *NSCLC* non-small cell lung carcinoma, *MVA* Modified Vaccinia Ankara, *OS* overall survival, *ORR* objective response rate, *PAP* prostatic acid phosphatase, *PD* progressive disease, PFS progression-free survival, PSMA prostate-specific membrane antigen, *Poly-ICLC* polyinosinic-polycytidylic acid polylysine carboxymethylcellulose, *PR* partial response, *SCLC* small cell lung cancer, *SD* stable disease, *TBI* total body irradiation, *TIL* tumor infiltrating lymphocyte, *TLPLDC* tumor lysate, particle-loaded, dendritic cell, *TLR* toll-like receptor, *VEGFR-2* vascular endothelial growth factor receptor-2, *WT-1* Wilms tumor gene-1

## Methodology

We performed a thorough review using the databases PubMed and the American Society of Clinical Oncology (ASCO), both the American Association of Cancer Research (AARC) meeting abstract databases, and ClinicalTrials.gov updated through October 5, 2017. We narrowed our research with the following keywords and MeSH terms: immunotherapy, tumor directed monoclonal antibodies, antibody drug conjugates, chimeric antigen receptor T-Cells, oncolytic viruses, oncologic vaccines, and adjuvants in immunotherapy. We focused our attention on phase I and phase II clinical trials of new agents in immunotherapy being used with or without other form of immunotherapy. Inclusion criteria included published trials or reported preliminary results during the time of the data collection. Exclusion criteria included phase III or more advanced clinical trials, clinical trials focusing only on immune checkpoint therapy, clinical trials in pediatric population, and non-clinical trials. Finally, we collected 65 phase I and 53 phase II clinical trials for this review.

## Tumor-directed monoclonal antibodies

A range of mAbs directed against tumor-specific antigens are currently under development. These mAbs can bind specific tumor antigens, stay on the surface, and activate antibody/complement-dependent cytotoxicity, or affect downstream signals. Monoclonal antibodies promote tumor killing by different mechanisms including direct cell killing by induction of apoptosis, receptor blockade or agonist activity, delivery of cytotoxic agents, radiation, immune-mediated cell killing, or through specific effects on tumor vasculature and stroma [4]. Of particular interest are the recently developed bispecific antibodies that combine antigen-binding specificities on tumor cells and effector immune cells. Among these, bispecific T cell engager (BiTE) and dual-affinity re-targeting (DART) are particularly attractive [5–7]. BiTEs recombinantly link the four variable domains of heavy and light chains with a flexible linker peptide allowing to bypass MHC/peptide recognition and co-stimulation and also to bring effector cells and target cells close together to form cytolytic synapses. This therapy has revealed impressive clinical activity in relapsed non-Hodgkin’s lymphoma, chronic lymphocytic leukemia, and acute lymphoblastic leukemia at doses much lower than those administered in conventional monoclonal antibody therapy. DART consists of a diabody that separates variable domains of heavy and light chains of the two antigen-binding specificities on two separate polypeptide chains stabilized through a C-terminal disulfide bridge which acts as a linker [5]. Compared with BiTE, DART has shown a moderately higher association rate constant for CD3 and an ability to cross-link T cells and B cells more efficiently [8, 9]. Ongoing clinical trials will provide more insightful understanding through side-by-side comparison of DART, BiTE, and other bispecific antibody with identical antigen-binding specificities. The quality, stability, and drug distribution of antibodies remain a challenge. Below outlines the ongoing early development of agents within this form of therapy.

Ensituximab (NPC-1C) is a chimeric IgG1 monoclonal antibody that promotes antibody-dependent cellular cytotoxicity after binding its target, a tumor-specific variant of MUC5AC, an antigen that is specifically expressed by colorectal (CRC) and pancreatic cancers. Results from a completed phase I/II clinical trial were recently published and are encouraging [10]. The therapy was well tolerated with < 2% of patients experiencing grade 3 toxicities and there were no grade 4 adverse events. Furthermore, median overall survival (OS) was significantly longer than historical control: 6.8 vs 5.0 months, 21 out of 56 patients survived > 12 months.

BiTEs simultaneously target two different antigens and thus target two different mediators and pathways [6]. CEA CD3 TCB (RG7802, RO6958688) is an IgG1 BiTE that simultaneously binds carcinoembryonic antigen (CEA) on tumor cells and CD3 on T cells to increase tumor-infiltrating lymphocytes (TIL) activation, infiltration, and expression of PD-1/PD-L1 [11]. Two ongoing phase I clinical trials using this new drug are currently recruiting patients (NCT02324257, NCT02650713). Preliminary results reveal that the most common adverse events were mild and 16% of patients develop grade 3 or more adverse events. Five percent and 20% of patients in each study, respectively, showed partial response (PR), and more importantly, activity appeared to be enhanced if it was combined with the anti-PD-L1 antibody, atezolizumab [11].

Blinatumomab is another BiTE that binds CD3 on T cells as well as CD19 on malignant B cells. The antibody is FDA-approved for the use of Philadelphia chromosome-negative B cell acute lymphoblastic leukemia (B-ALL) [12]. A phase II clinical trial in relapsed/refractory diffuse large B cell lymphoma demonstrated a complete response (CR) rate of 19% and progression-free survival (PFS) of up to 20 months [13].

BAY2010112 (AMG212, MT112) and MOR209/ES414 are prostate-specific membrane antigen (PSMA)/CD3 BiTEs that are being investigated in phase I clinical trials in patients with castration-resistant prostate cancer (NCT01723475, NCT02262910).

DARTs differ structurally to BiTEs as stated above [5]. MGD009 is a humanized DART protein that binds both T cells and tumor-associated B7-H3 and is being studied in a phase I clinical study in patients with B7-H3 expressing tumors including melanoma, non-small cell lung carcinoma (NSCLC), mesothelioma, and urothelial cancers [14]. The trial is ongoing and recruiting patients (NCT02628535). Flotetuzumab, another DART that binds CD3 and CD123, is currently being studied in a phase I clinical trial in patients with relapsed or refractory acute myeloid leukemia (AML) and intermediate/high-risk myelodysplastic syndrome (MDS) (NCT02152956).

AFM13 is a tetravalent bispecific antibody that is directed against CD30 and CD16A, this latter found over natural killer (NK) cells. Pharmacokinetics, therapeutic index, and efficacy make this agent a NK cell activator [15]. A phase I clinical trial used AFM13 on patients with relapsed/refractory CD30+ Hodgkin’s disease (HD) and concluded that three out of 26 patients achieved PR and 13 obtained a SD with an overall disease control rate (DCR) of 61.5% [16]. Adverse events were mostly mild to moderate and ranged from fever to infusion reactions and pneumonia. A phase II clinical trial using this agent is also being done on patients with HD; however, results have not yet been published (NCT02321592).

## Antibody drug conjugates

Antibody drug conjugates (ADCs), an emerging therapeutic approach in oncology, combine a monoclonal antibody with a high selectivity for specific targets with a cytotoxic agent. Microtubule inhibitors or DNA-damaging chemotherapeutic agents are the two main cytotoxic agents used in ADCs. One of the most important aspects in ADC therapy consists on the appropriate selection of the target antigen. An ideal antigen is one that is overexpressed by malignant cells with very limited or no expression by normal tissue [17]. For example, nectin-4 is often overexpressed in bladder, breast, lung, and pancreatic cancer, and thus, ACDs against this peptide are indicated in these malignancies. Similarly, folate receptor alpha is more often expressed by ovarian and endometrial carcinomas, and CEA cell adhesion molecule (CEACAM) 5 is commonly found on CRC [17]. Another important factor is the conjugate linker, which largely influences the pharmacokinetics and therapeutic efficacy of ADCs [17]. As with other drugs, resistance can emerge through different mechanisms such as limitation of intracellular concentration of ADC, downregulation of target antigens, reduction of internalization of ADC, increased ADC recycling to mask the antigen epitopes, or activation of alternative signal pathways. Class side effects are often associated with the linked cytotoxic agents. The major challenges of ADCs include target antigen specificity and drug delivery efficiency.

ABBV-399 is an ADC composed of an anti-c-Met antibody (ABT-700) conjugated to a microtubule inhibitor (monomethyl auristatin E). As the c-Met receptor is commonly overexpressed in patients with NSCLC, a phase I clinical trial is using this agent as monotherapy or in combination with erlotinib in this patient population (NCT02099058). Preliminary results demonstrated adverse events in > 10% of patients across all grades, 19% of patients (3 out of 16) had a PR, and, at week 12, 37.5% (6 out of 16) had disease control including stable disease and partial response [18].

Glembatumumab vedotin (GV, CDX-011) is an ADC that contains an antibody that targets glycoprotein non-metastatic b (gpNMB), a transmembrane glycoprotein usually overexpressed in melanoma and other tumors, conjugated to monomethyl auristatin E. A phase II clinical trial using this agent as monotherapy in patients with advanced melanoma is recruiting patients (NCT02302339). Preliminary results show 1 CR, 6 PR, and 33 SD out of 62 patients enrolled [19].

Losatuxizumab vedotin (ABBV-221), an ADC that targets EGFR, is being investigated in a phase I clinical trial as monotherapy on patients with EGFR-dependent tumors (NCT02365662). Preliminary results reveal that the most common adverse events were infusion reactions and fatigue and 16 out of 42 patients (38%) showed SD and 1 patient had an unconfirmed PR [20].

Mirvetuximab soravtansine (IMGN853) is an ADC containing the tubulin inhibitor (maytansinoid) DM4, targeting the folate receptor alpha (FRα). It is being studied in two phase I clinical trials as monotherapy (FORWARD I-NCT01609556) and in combination with bevacizumab, carboplatin, liposomal doxorubicin, or pembrolizumab (FORWARD II-NCT02606305) in patients with ovarian cancer. Preliminary results of FORWARD I reveal an objective response rate (ORR) of 46% with one patient having a CR and 16 out of 37 patients having PR [21]. FORWARD II preliminary results do not mention efficacy but do show a good safety profile with most adverse events being grade 2 or less [21].

Enfortumab vedotin (ASG-22CE; ASG-22ME), an ADC that targets nectin-4, is in a phase I clinical trial that is investigating its potential role as monotherapy in patients with metastatic urothelial tumors (NCT02091999). Preliminary results show that adverse events occurred in 85% of patients; however, most were grade 2 or less. ORR was 40%, CR was seen in 3 out of 68 patients with a median duration of response of 18 weeks, and the median PFS was 17 weeks [22].

Sacituzumab govitecan (IMMU-132) is an ADC against Trop-2 antigen expressed in many solid tumors and carrying the topoisomerase inhibitor, SN-38. A phase I/II clinical trial as monotherapy in patients with epithelial cell tumors is undergoing (NCT01631552). Preliminary results in triple negative breast cancer show the agent is well tolerated demonstrating an ORR of 30% with two cases of CR and 19 out of 69 patients demonstrating a PR. Median PFS was 6 months and median OS was 16.6 months [23]. In NSCLC, an ORR of 19% was observed among the 47 patients, with a median response duration of 6 months, a median PFS of 5.2 months, and a median OS of 9.5 months [24].

Inotuzumab ozogamicin (InO/CMC-544) is a humanized ADC directed against CD22, coupled to a DNA breaking calicheamicin. The compound was recently approved by the FDA for use in relapsed or refractory B-ALL [25]. This agent was recently evaluated in a phase I clinical trial in conjunction with rituximab, gemcitabine, dexamethasone, and cisplatin in patients with refractory CD22+ non-Hodgkin’s lymphoma (NHL) [26]. Results demonstrated an 85% incidence of thrombocytopenia and a 69% of neutropenia with an ORR of 53%.

Labetuzumab govitecan (IMMU-130) is an ADC that targets CEACAM 5 which is expressed by > 80% of CRC [27]. This ADC is being evaluated in a phase II clinical trial in patients with metastatic CRC (NCT01605318). Results reveal that one out of 86 patients enrolled had a PR that extended beyond 2 years, 42 patients had SD, OS was 6.9 months, and PFS was 3.6 months [27].

Lorvotuzumab mertansine (IMGN901), an ADC against CD56 conjugated to the tubulin inhibitor DM1, that was recently studied in a phase I/II clinical trial in combination with carboplatin and etoposide in small cell lung cancer (SCLC) patients with extensive disease [28]. The two-arm cohort of 94 patients (combination ADC with chemotherapy) and 47 patients (no ADC) achieved a PFS of 6.2 and 6.7 months, respectively, with a median OS of 10 months in both cohorts. The ORR was 67% for the combination cohort compared to 59% in the non-combination with no statistical significance. Thus, the authors concluded that the combination of lorvotuzumab mertansine did not improve efficacy over standard therapy [28].

Rovalpituzumab tesirine (Rova-T) is an ADC that targets delta-like protein 3 (DLL3) which has been found to be elevated in patients with SCLC. The antibody is conjugated to a DNA cross-linker tesirine, and results from the first-in-human trial in patients with recurrent SCLC showed that 28 out of 74 patients (38%) developed serious adverse events consisting of pleural and pericardial effusions, 18% demonstrated OR, and 54% had SD [29]. Other phase I/II clinical trials evaluating this agent are currently ongoing (NCT02674568, NCT03026166).

ADCT-301 is the first ADC against CD25, a receptor for IL-2 often found on hematological tumors that has a role in prognosis and oncogenesis in these malignancies [30]. This molecule is being studied in a phase I clinical trial in patients with relapsed or refractory HD and NHL (NCT02432235). Preliminary results on 18 patients show 1 CR, 1 PR, and 6 SD, one of which has remained progression-free for over 30 weeks and four developing adverse events consisting of rash, mucositis, enteritis, and elevated creatine phosphokinase [31]. Another phase I clinical trial is being conducted in patients with refractory or relapsing CD25-positive AML and ALL (NCT02588092).

TAK-264 (MLN0264), a novel ADC that targets guanylyl cyclase C (GCC), has been recently studied in a phase II clinical trial in patients with advanced or metastatic pancreatic adenocarcinoma expressing GCC [32]. The cohort of 43 patients achieved an ORR of only 3%, and all patients experience at least one adverse event; thus, the authors concluded these results did not support further clinical investigation of this molecule.

HER2, a well-studied member of the epidermal growth factor tyrosine kinase receptor family, plays an important role in breast cancer and has been a target for ADCs. T-DM1 (Kadcyla), an ADC consisting of trastuzumab (T) and a microtubule inhibitor (DM1), was the first ADC approved by the FDA to use in solid tumors [33]. Other anti-HER2 ADCs are being studied in multiple phase I/II clinical trials including MEDI-4276 (NCT02576548), XMT-1522 (NCT02952729), ARX-788 (NCT02512237), DS-8201a (NCT02564900), SDY985 (NCT02277717), and ADCT-502 (NCT03125200).

Glycoprotein non-metastatic b (GPNMB), a highly expressed protein in melanoma and breast cancer, plays an important role with modulation, and is an important target in ADC. Glembatumumab vedotin (CDX-011, CR-11-vc-MMAE) targets GPNMB, and a detailed review on this agent has been recently published [34]. A phase II clinical trial on patients with melanoma (NCT02302339) demonstrated that, while using this ADC, 1 out of 62 patients achieved CR, 6 achieved PR, and 33 had SD, and the median OS was 9.8 months. Toxicities were manageable and included alopecia, neutropenia, and rash [19]. Another phase II clinical trial using glembatumumab vedotin on patients with advanced breast cancer demonstrated an ORR of 6% with mild toxicities including rash and pruritus [35].

CA6 is a tumor-associated antigen that can be overexpressed in many solid tumors with a low expression on normal tissues [33]. SAR566658 targets CA6 and delivers maytansinoid DM4. On a first-in-human clinical trial on patients with solid tumors expressing CA6 using this agent, 114 patients were enrolled. Results revealed 1 CR, 8 PR, and 39% SD with overall mild toxicities including fatigue, neuropathy, neutropenia, and gastrointestinal symptoms [36].

LIV-1 is a transmembrane protein that is highly expressed in breast cancer cells. SGN-LIV1A is an anti-LIV-1 ADC that is being tested on a phase I clinical trial on patients with metastatic LIV-1-postive breast cancer (NCT01969643). Preliminary results demonstrate no dose-limiting toxicities, an ORR of 11% and an SD or better achieved in 63% of patients [37].

PF-06647020 is an ADC directed against protein tyrosine kinase 7 (PTK7) which is overexpressed in a variety of tumors including lung, CRC, breast, and ovarian cancers [33]. This molecule is being studied in a phase I clinical trial on patients with advanced solid tumors (NCT02222922). Preliminary results revealed that 1 out of 76 patients had a CR, 5 had PR, 12 had SD, and 4 had PD. Most toxicities were grades 1–2 and included nausea, alopecia, neutropenia, and gastrointestinal complaints [38].

Ephrin-A4 has been found to be elevated in multiple malignancies including lung, pancreas, and breast [33]. PF-06647263 is an anti-Ephrin-A4 ADC that is coupled to calicheamicin and that was studied in a phase I clinical trial on patients with advanced solid tumors [39]. Results revealed that 5 out of 48 patients achieved PR and that dose-limiting toxicities were observed in 6 patients [39].

LAMP-1 is a protein highly expressed by lysosomes that translocates to the surface of tumor cells, and its expression may influence invasiveness and metastatic behavior including CRC, melanoma, and laryngeal cancers [33]. SAR428926 is an anti-LAMP-1 ADC coupled with DM4 that is currently being studied on a phase I clinical trial in patients with advanced solid tumors (NCT02575781). Preliminary results have not been published.

PCA062 is another ADC that is targeted against P-cadherin 3, which is often overexpressed in epithelial tumors [33]. This agent is being studied in a phase I clinical trial on patients with P-cadherin positive tumors (NCT02375958). No preliminary results have been published.

HER-3 overexpression in breast cancer is associated with poor prognosis, and an anti-HER3 ADC therapy, U3-1402, is being studied in a phase I clinical trial on patients with HER-3-positive metastatic breast cancer (NCT02980341) [40]. Preliminary results have not been published. HuMax-Axl is an ADC against an Axl-specific immunoglobulin (IgG1κ) which is present in malignancies like pancreatic, thyroid, and lung cancer and melanoma [41]. A phase I/II clinical trial on patients with solid tumors is studying the use of this agent (NCT02988817); however, preliminary results are not yet available.

PSMA is highly expressed in prostate cancer, and MEDI3726, an anti-PSMA ADC, is being studied on a phase I/Ib clinical trial in patients with metastatic castration-resistant prostate cancer (NCT02991911). Preliminary results are yet to be published.

## Chimeric antigen receptor (CAR) T cells

CARs are typically genetically engineered T cell receptors with an antibody-based extracellular domain that specifically recognizes a tumor antigen, a transmembrane portion, and an intracellular domain that activates the T cell. By antigen-specific recognition in a MHC-independent manner, CAR T cells are activated in vivo through phosphorylation of immune receptor tyrosine-based activation motifs (ITAMs) leading to cytokine secretion, T cell proliferation, and antigen-specific cytotoxicity. CAR T cells are produced by inserting specific CAR genes via viral vectors into autologous or allogeneic T cells [42]. New-generation CARs have two or more co-stimulatory domains (e.g., 4-1BB, OX 40) that boost the stimulatory signal [43, 44]. Anti-CD19 CAR T cells were recently FDA-approved for B-ALL in pediatric and young adult population [45]. Other CARs rely on the ligand of the receptor of interest rather than on an antibody [46]. Although impressive clinical activities of CAR T cells in hematological malignancies are reported, several obstacles need to be overcome for a successful application of CAR T cells in solid tumor [47]. Some of these obstacles include the lack of ideal tumor-specific antigens, the inefficient trafficking of CAR T cells to tumor sites, the immune-suppressive tumor microenvironment, and the risk of developing on-target/off-tumor toxicities which results from an attack to host cells that express the targeted tumor antigen. One of the major challenges with this therapy is the cytokine release syndrome (CRS). This potentially fatal toxicity occurs after a massive release of cytokines and causes complications ranging from mild fever and fatigue to severe respiratory distress, cardiac dysfunction, or even disseminated intravascular coagulation [48]. Future investigation to improve the safety, specificity, and efficiency will likely take CAR T cell therapy into the central stage in cancer immunotherapy [49].

### T4 immunotherapy

T4 immunotherapy uses genetically engineered T cells that co-express two CARs, T1E28z that targets ErbB dimers, and 4αβ that binds IL-4 and promotes T cell expansion [50]. These CAR T cells are currently undergoing a phase I clinical trial on patients with head and neck squamous cell carcinoma (HNSCC) (NCT01818323). Preliminary results show limited adverse events and a disease control rate (DCR) of 44% [51].

### Anti-CD19 CAR T cells (CART-19)

19-28z CAR (JCAR015) consists of a single-chain murine antibody against human CD19 (expressed by B cell malignancies) fused with the transmembrane and cytoplasmic domains of the human CD28 co-stimulatory molecule [52]. Currently, these CAR T cells have been studied in a phase I clinical trial in patients with relapsed B-ALL (ROCKET Trial-NCT01044069). In this trial, patients were divided into two cohorts, those with < 5% of blasts in the bone marrow and those with > 5%. CR rates were 95 and 77%, respectively. The duration of this response, however, was directly proportional to the disease burden, and therefore, authors suggest an early use of this therapy before > 5% of blasts occupy the bone marrow [53].

The ZUMA-1 trial is studying anti-CD-19 CAR T cells (KTE-C19) in patients with refractory aggressive NHL (NCT02348216). Preliminary results reveal an ORR of 82%, and with a median follow-up of 8 months, 39% remained in CR. Importantly, 43 patients received tocilizumab and 27 out of 111 received steroids; however, ORR did not change significantly with the addition of this therapy [54].

CART-19 are also being studied in other malignancies including Richter syndrome [55] and relapsed chronic lymphocytic leukemia, myeloma, or NHL (NCT01865617). Although in one study 4% died due to CRS [56], efficacy results seem promising with some patients achieving CR or remission at the time of result publication [56, 57].

### Anti-GPC3 CAR T cells

Glypican-3 (GPC3) is a membrane proteoglycan selectively expressed by hepatocellular carcinoma (HCC) cells that is being used as a target CAR therapy [58]. GPC3 CAR T cells were studied in a phase I clinical trial of patients with GPC3+ HCC. Preliminary results showed no dose-limiting toxicities; 1 out of 13 patients had a PR, and 3 showed SD. These responses were seen in patients who received lymphodepletive conditioning [59].

### Anti-CD133 CAR T cells (CART-133)

CD133 is expressed by many tumors of epithelial origin, and therefore, the use of CAR T cells against this molecule (CART-133) is undergoing investigation being in a phase I clinical trial in patients with advanced metastatic malignancies including HCC, pancreatic carcinoma, CRC, and cholangiocarcinoma (NCT02541370). Preliminary results show some grade 3 adverse events consisting of hyperbilirubinemia and CRS. However, 21 out of 23 patients had PFS periods ranging from 8 to 22 weeks. Furthermore, 2 patients maintained greater than 8-month SD at the time of publication [60].

### Anti-BCMA CAR T cells (bb2121)

B cell maturation antigen (BCMA) is expressed by multiple myeloma (MM) cells, and anti-BCMA CARs have been genetically incorporated to T cells [61]. One of these CAR T cells, bb2121, is currently under investigation in a phase I clinical trial on patients with refractory MM who have ≥ 50% BCMA expression on their plasma cells (NCT02658929). Preliminary results show only minor adverse events with grade 1 or 2 CRS, and an ORR was seen in all patients (6 out of 11) who received the higher doses of T cells [62].

LCAR-B38 is another CAR T cell that targets BCMA that was recently studied in a phase I clinical trial of patients with refractory or relapsed MM. Preliminary results were encouraging. Seventy-four percent of patients (14 out 19) developed CRS, but most of these were mild in severity. Importantly, ORR and CR/near CR rates were 100 and 95%, respectively, at a median follow-up of 6 months [57].

### Anti-CD138 CAR T cells

CD138 is a highly expressed molecule on MM cells and has a role in their development and proliferation [63]. A phase I clinical trial using anti-CD138 CAR T cells was conducted in patients with chemotherapy-refractory MM [63]. Five patients were enrolled, of which 4 achieved SD for more than 3 months and 1 with PD. The main toxicity observed was CTS.

### Anti-immunoglobulin kappa light chain CAR T cells

As a way to spare normal cells from being targeted by this therapy, CAR T cells directed against more tumor-specific proteins like kappa light chains are also being developed [64]. A phase I clinical trial testing this therapy on patients with kappa-positive chronic lymphocytic leukemia (CLL), NHL, or MM is currently undergoing (NCT00881920). Results of this trial showed that 2 out of 9 patients with NHL or CLL had CR and 1 had PR. Four of the 7 patients with MM showed SD that lasted 2–17 months. No toxicities were seen with this therapy [64].

### Anti-CD30 CAR T cells

CD30 is expressed in a limited amount on normal tissues whereas is often overexpressed in patients with HD and NHL [65]. The use of anti-CD30 CAR T cells was recently studied in a phase I clinical trial on 9 patients with refractory or relapsed HD and anaplastic large cell lymphoma (ALCL) [66]. No toxicities were observed with this therapy, 2 patients with HD and 1 with ALCL achieved CR, and 3 patients with HD achieved SD. The use of anti-CD30 CAR T cells in combination with bendamustine is being studied on a phase Ib/II clinical trial on patients with CD30+ HD and NHL (NCT02690545).

### Anti-IL13 CAR T cells

Recently, the use of CAR T cells targeting IL13Rα2 in a patient with recurrent multifocal glioblastoma demonstrated CR sustained for 7.5 months with no associated systemic side effects [67].

### CARs on the horizon: NKG2D, NKR-2, and mesothelin CAR T cells

Natural killer group 2D (NKG2D) receptor recognizes specific ligands over tumor cells and promotes T cell activation to eliminate NKG2D ligand-expressing cells [68]. A first-in-human phase I clinical trial is using genetically modified T cells expressing NKG2D in patients with AML, MDS, and MM (NCT02203825).

NKR-2 CAR T cells consist of a fusion of NKG2D receptor with CD3 signaling domain, which are under investigation on a phase I clinical trial in patients with both solid and hematologic malignancies (THINK–Therapeutic immunotherapy with NKR-2–trial) (NCT03018405).

Finally, mesothelin as a target for CAR T therapy in patients with mesothelioma has been recently reviewed [69]. Various phase I clinical trials are exploring treatment approach (NCT02414269, NCT01583686, NCT02580747). Results for these future studies have yet to be reported.

## T cell receptor (TCR) gene-modified T cell therapy

In contrast to CAR T cell therapy, TCR gene-modified T cell therapy functions by targeting the surface antigens of tumor cells to specifically recognize intracellular tumor antigens presented by HLA molecules. By genetic transfer of TCR directed against specific tumor antigens into normal T cells, T cells are able to perform antigen-specific tumor killing. In addition, genetic engineering of T cell also offers the advantage of introducing molecules that can enhance T cell function or overcome tumor escape mechanisms, such as adding genes encoding cytokines, chemokine receptors and costimulatory factors, as well as elements to silence inhibitory molecules. Currently, genes encoding TCRs that are specific for a variety of tumor antigens such as MART-1, gp100, p53, NY-ESO-1, MAGE-A3, and MAGE-A4 have been studied as therapeutic targets for TCR gene-modified T cell therapy in clinical trials for melanoma, lung cancer, and breast cancer patients [70]. Choosing highly specific tumor antigens, maintaining TCR expression over time, and limiting off tumor/on target toxicity are remaining challenges [70].

### Anti-NY-ESO-1 TCR T cells (NY-ESO-1c259t)

NY-ESO-1 is expressed in up to 70% of synovial sarcomas and other mesenchymal tumors and the use of TCR gene-modified cells, specifically, NY-ESO-1c259T cells that recognize an HLA-A2 peptide are being studied. An ongoing phase I/II clinical trial is recruiting patients with unresectable or metastatic synovial sarcoma who express NY-ESO-1 and HLA-A2 (NCT01343043). Preliminary results demonstrated the development of leukopenia (96%), anemia (79%), thrombocytopenia (79%), one fatal bone marrow failure, and 11 out of 34 cases with CRS. However, ORR was 50% and 1 patient had a PR [71].

### Anti-E6 TCR T cells

The E6 oncoprotein is an essential component of HPV-related tumorigenesis, and its expression is maintained in advanced lesion and represents an ideal tumor-specific antigen. TCR gene-modified T cells that express a TCR that recognize an HLA-A*02:01-restricted epitope of E6 has been developed [72]. A phase I/II clinical trial using these T cells in patients with metastatic HPV 16+ carcinomas, including cervical, anal, and pharyngeal, was recently completed. No dose-limiting toxicity was seen, and 2 out of 12 patients demonstrated PRs [73].

### Anti-MAGE A10 TCR T cells

MAGE-A10 peptide is expressed by different malignancies including urothelial, HNSCC, and melanoma [74]. The use of T cells containing the MAGE-A10c796 CAR is being studied in a first-in-human phase I clinical trial on patients with advanced or inoperable urothelial cancer, HNSCC, or melanoma (NCT02989064). Preliminary results are not available yet.

## Tumor-infiltrating T cell therapy

Adoptive cell therapy that utilizes endogenous tumor-infiltrating lymphocytes (TIL), which are expanded in vitro from a surgically resected tumor and then re-infused back into the patient, has demonstrated a 20% complete response lasting beyond 3 years in patients with stage IV melanoma [75]. TILs are naturally occurring T cells in the host able to recognize tumor antigens. This likely explains the highly specific anti-tumor responses and the relatively low toxicity of TILs in comparison with TCR gene-modified T cell therapy and CAR T cell therapy. In addition, TILs are heterogeneous in their specificity which represents an important advantage for impeding immunologic escape. Furthermore, TIL therapy bypasses the limitation identifying specific tumor antigens or the patient’s HLA type. Nonetheless, all its clinical advantages are somewhat blunted by the complex process required to generate patient-specific TILs for clinical use. Strategies to improve and simplify the TIL production are being studied.

Infusion of ex vivo expanded TIL is currently being studied in conjunction with total body irradiation (TBI) in a phase II clinical trial in patients with melanoma (NCT01319565). Preliminary results reveal that 13 out of the 48 patients who received TBI developed thrombotic microangiopathy, but this was not seen in the group without TBI. Regardless, a CR rate was seen in 24% of patients in both groups, and only one of these patients had recurrence of the disease [76].

Marrow-infiltrating lymphocytes (MILs) have been used in patients with newly diagnosed or relapsed MM. In a phase I clinical trial, the overall clinical response was of 54%, with 27% CR and 27% PR, 23% of patients had a SD, and only 14% showed a PD. Patients who achieved at least 90% reduction of disease burden had a PFS almost 13 months longer; however, no difference in OS was seen [77].

## Oncolytic viruses

Native or genetically modified viruses are a new therapeutic approach within the immunotherapy spectrum. The mechanisms of action of oncolytic viruses are not fully elucidated but likely depend on viral replication within tumor cells, induction of primary cell death, interaction with tumor cell antiviral elements, and initiation of innate and adaptive anti-tumor immunity. A variety of native and genetically modified viruses have been developed as oncolytic agents [78]. Of note, these viruses selectively infect malignant cells due to the lack of adequate function of anti-viral mechanisms. Though many viruses have been considered, the most widely studied to date include herpes simplex virus type 1 (HSV-1), coxsackievirus, reovirus, and adenovirus.

Talimogene laherparepvec (T-VEC; Imlygic) is the first oncolytic virus approved by the FDA for its use in melanoma. It is an attenuated HSV-1 engineered to replicate within tumor cells and enhance immune responses [79]. Treatment has been relatively well tolerated, with the major side effects including fever, chills, nausea, fatigue, and reaction of local injection site.

Coxsackievirus A21 (CVA21–CAVATAK) preferentially infects tumors that express ICAM-1. This virus increases TIL and PD-L1 expression [80]. Therefore, the use of CVA21 is currently being studied in conjunction with pembrolizumab in two phase Ib clinical trials in patients with solid tumors including melanoma, NSCLC, bladder, and prostate cancer (NCT02043665, NCT02565992). Both STORM [81] and CAPRA [82] trials demonstrated that the combination is generally well tolerated and the latter also showed an ORR of 73% and a DCR of 91% of patients with advanced melanoma. CVA21 is also being studied in conjunction with ipilimumab in patients with unresectable melanoma (NCT02307149). Preliminary results demonstrate minimal additional toxicity with an ORR of 38% (3 out of 8 patients) and a DCR of 88% (7 out of 8 patients) [83].

Pelareorep (Reolysin) is a strain of reovirus serotype-3 which has shown in vitro and in vivo activity against many cancers and synergistic activity with concomitant use of microtubule-targeting drugs [84]. This agent has been studied in a phase II clinical trial in combination with carboplatin and paclitaxel for patients with advanced malignant melanoma. Results revealed and ORR of 21%, no CR, and a 1-year survival of 43% with a DCR of 85% [85]. Another phase II clinical trial using pelareorep with paclitaxel versus paclitaxel alone in patients with metastatic breast cancer was also recently published [84]. Median OS was 17.4 months for patients with both agents and 10.4 months for patients with paclitaxel alone; however, PFS was not different between the groups.

The oncolytic adenovirus DNX-2401 was also studied in a phase I clinical trial using temozolamide in patients with first recurrence of glioblastoma [86]. One patient out of 31 was still alive 30 months after the treatment was started, and 2 other patients were still alive 23 months after the agent was given.

Enadenotucirev (EnAd) is an A11/Ad3 chimeric group B oncolytic adenovirus that is currently under investigation in combination with nivolumab for patients with tumors of epithelial origin such as salivary gland, urothelial, HNSCC, and CRC (NCT02636036). No preliminary results have yet been published.

## Vaccines

Therapeutic vaccines are designed to increase immune response against malignant cells by enlarging antigen-specific T cell from endogenous T cell repertoire. Its use has the advantage of producing a specific immune response that potentially spares normal cells [87]. Successful cancer vaccines require the selection of appropriate antigens, a platform able to induce robust effector and memory T cell responses, and strategies to overcome immune evasion and suppression. Although cancer vaccines can be effective in settings of early cancer or minimal residual disease, therapeutic cancer vaccines will most likely require co-treatment, such as immune checkpoint inhibitors, to overcome immune suppression and be clinically effective in established cancers.

Depending on its composition, vaccines can be classified into tumor cell vaccines (autologous/allogenic), genetic vaccines (DNA/RNA/viral/bacterial), dendritic cell (DC) vaccines, and protein/peptide vaccines.

### Tumor cell vaccines

Tumor cell vaccines are classified as autologous when patient-derived tumor cells are used, or allogenic if established human tumor cell lines are the utilized. Among the allogenic vaccines, HS-110 (viagenpumatucel-L) is derived from lung adenocarcinoma cells. This vaccine is currently being studied in combination with nivolumab on a phase I/IIb clinical trial (DURGA trial) which is recruiting patients with NSCLC (NCT02439450). Preliminary results showed ORR of 50% in patients with IR (immune response, defined by doubling of IFNγ-secreting cells after re-stimulation with total vaccine antigen and individual cancer antigens) in comparison to 0% in patients without immune response among the initial treated 8 patients. Interestingly, patients with objective responses were also found decrease in MDSC and increase in CD8+ T cells in the blood [88].

An autologous tumor-derived gp96 vaccination was studied in a phase II clinical trial in patients with advanced gastric cancer [89]. The trial enrolled 73 patients of which 38 received both vaccination and chemotherapy and the remainder received chemotherapy alone. Overall vaccination was well tolerated with no clinically significant adverse events. The 2-year OS was 81.9% in the vaccination group compared to 67.9% in the chemotherapy-alone arm, though this was not statistically significant [89].

The GM.CD40L is another allogenic vaccine composed of radiated lung adenocarcinoma cells transduced with the GM-CSF and CD40-ligand (CD40L) genes. This vaccine is being studied in patients with lung adenocarcinoma in a phase I/II clinical trial in combination with nivolumab (NCT02466568) and in combination with CCL21, a chemokine that enhances T cell response (NCT01433172). Preliminary results reveal an acceptable safety profile with no dose-limiting toxicities and a median OS similar to single-agent nivolumab (9.4 months) [90].

### Genetic vaccines

Genetic vaccines use DNA/RNA plasmids, bacteria, or viruses to deliver antigens by transfection of cells that subsequently process them and present them to immune cells [91].

The use of messenger RNA (mRNA) that encodes tumor antigens is currently being studied in clinical trials. RNA-Lipoplex (RNA_(LIP)_) is a mRNA vaccine that encodes melanoma antigens (e.g., NY-ESO-1). It is being studied in a first-in-human phase I/II (Lipo-MERIT) clinical trial (NCT02410733). Preliminary results in 15 patients show no dose-limiting toxicities, but no efficacy results have been published yet [92].

VXM01 is an oral vaccine derived from live, attenuated Salmonella carrying a DNA plasmid that encodes vascular endothelial growth factor receptor-2 (VEGFR-2). A phase I clinical trial using this vaccine in patients with advanced pancreatic carcinoma showed that 12 out of the 18 patients studied had a considerable increase of specific anti-VEGFR2 T cells and the OS was 9.3 months compared to 8.4 months in those who received placebo. Furthermore, patients who showed T cell response had a longer median OS (10.3 months) compared to those without it (5.4 months) [93].

INO-5150 is a plasmid-based DNA vaccine that encodes for highly expressed prostate cancer antigens with amino acid sequence changes to break immune tolerance. This vaccine is being investigated with and without co-administration of IL-12 (INO-9012) in a phase I clinical trial (NCT02514213). Preliminary results revealed no dose-limiting toxicities and PSA was stable in some patients, whereas 10% reported disease progression [94].

INVAC-1 is another plasmid DNA vaccine that encodes an inactive form of human telomerase, which is expressed in over 85% of human tumors [95]. This vaccine is currently being studied in a phase I clinical trial with refractory and progressive solid tumors (NCT02301754). Preliminary results reveal no dose-limiting toxicities with only mild adverse events, 12 out of 20 patients achieved SD, and anti-human telomerase activity was found in 55% of patients [95].

pTVG-HP, also a plasmid DNA vaccine, encodes prostatic acid phosphatase (PAP). It is being studied in a phase II clinical trial in patients with non-castrate, non-metastatic prostate cancer (NCT01341652).

*Listeria monocytogenes* can survive in the cytosol of host cells and is therefore considered an ideal vector for tumor antigens [96]. ADXS11-001 is a live, attenuated *Listeria monocytogenes*, which is bioengineered to secrete a HPV-E7 antigen, a marker of HPV transformed cells. This vaccine is currently being investigated in a phase I clinical trial in patients with persistent, recurrent, or metastatic cervical carcinoma (NCT02164461). Preliminary results revealed that most adverse events were mild (grades 1 and 2), with no grade 4 or 5 reported. Tumor response analysis is ongoing and not yet published [97].

Adenovirus is also used as a vaccine vector. In a phase I clinical trial, the use of the cancer antigen MAGE-A3 primed to an adenovirus (AdMA3) is being investigated in conjunction with an oncolytic virus (MG-1 Maraba virus) that also expresses the MAGA-A3 antigen (MG1MA3), in patient with MAGE-A3 expressing solid tumors (NCT02285816). Preliminary results reveal a potent induction of pro-inflammatory genes with subsequent anti-tumor activity. However, dose-limiting toxicities also occurred in 4 out of 41 patients manifested by hypoxia, dyspnea, vomiting, and headache [98].

### Dendritic cell vaccines

DCs play an important role in bridging innate and adaptive immunity, and as such are considered an important target for immunotherapy. The autologous tumor lysate, particle-loaded, dendritic cell (TLPLDC) vaccine consists of DCs that are exposed to autologous tumor antigens, become particle-loaded, and are infused back to the patient. This vaccine is being investigated in patients with stage III or IV of ovarian cancer and preliminary results reveal minimal toxicity, 1 out of 12 patients demonstrated CR, 1 had a SD, and 4 had a progression of their disease [99]. AdHER2ECTM consists of autologous DCs expressing human HER2 extracellular and transmembrane domains and is currently under investigation in a phase I clinical trial on patients with advanced tumors expressing HER-2 (e.g., colon, breast, ovarian) (NCT01730118). Preliminary results show that adverse events were limited to local injection site reactions, 37% had evidence of response, namely, 1 out of the 27 evaluated patients demonstrated CR, 1 had PR, and 5 had SD [100].

CMB305 is another DC vaccine that carries the NY-ESO-1 gene and a boost with G305, a NY-ESO-1 protein vaccine [101]. This vaccine is currently being investigated in a phase I clinical trial in patients with NY-ESO-1-expressing solid tumors. Preliminary results reveal an increase of anti-NY-ESO-1 T cells in up to 65% of patients and anti-NY-ESO-1 antibodies in up to 68% of patients [101].

A Modified Vaccinia Ankara (MVA) vector vaccine expressing genes for brachyury (a transcription factor important in the epithelial-to-mesenchymal transition and in tumor resistance to treatment) and costimulatory molecules (e.g., ICAM-1) designated TRICOM was developed for transducing DCs [102]. This vaccine was studied in a phase I clinical trial in 38 patients with advanced solid malignancies [102]. No dose-limiting toxicities were observed, and 82% of patients developed brachyury-specific immune responses.

BPX101 is another DC-derived vaccine which was recently evaluated in a phase I clinical trial in 18 men with progressive metastatic castrate-resistant prostate cancer [103]. Results revealed no dose-limiting toxicities, one case of CR, and two PR.

### Protein/peptide-based vaccines

The use of specific tumor-associated antigens has the advantage of inducing specific immune response against defined antigens, sparing healthy tissue. However, because only defined epitopes are used, a specific but sometimes insufficient response may be generated as tumor cells often exhibit mutations of the epitopes used [104].

Wilms tumor gene-1 (WT-1) is overexpressed in many hematological and solid malignancies where it plays an oncogenic role [105]. The use of WT-1 peptide vaccine with gemcitabine was investigated in a phase II clinical trial on patients with pancreatic adenocarcinoma [106]. Results revealed an increased OS from 21.5 to 34.2 months in patients with gemcitabine alone compared to gemcitabine and WT-1 peptide vaccine, respectively. WT4869 is another peptide vaccine derived from WT1 that was recently studied in a phase I/II clinical trial in 26 patients with MDS. Results reveal an ORR of 18.2% and a median OS of 64.71 weeks, with an anti-WT1 lymphocyte induction occurring in 11 patients [107].

Galinpepimut-S is a WT-1-derived peptide vaccine with GM-CSF and Montanide as adjuvants that is being studied with compared to these last two alone, in a phase II clinical trial in 41 patients with pleural mesothelioma [108]. Results revealed a PFS of 45% in the patients with the vaccine arm compared to 33% in those without the vaccine. Median OS was 22.8 months in the vaccine group compared to 18.3. Most adverse events were not clinically significant. Unfortunately, the trial did not achieve statistical power.

DPX-Survivac is a peptide-based vaccine containing survivin epitopes to elicit cytotoxic T cell response against survivin-expressing tumors. A phase Ib clinical trial on patients with ovarian/fallopian/peritoneal cancer studied this vaccine in combination with low-dose of cyclophosphamide [109]. Besides being safe, it demonstrated achieving sustained immune responses. This vaccine is also being investigated in hematologic malignancies in a phase II clinical trial that is currently recruiting patients (NCT02323230).

AE37 is a vaccine that contains HER-2-derived epitopes that stimulate T cell response. It is currently being studied on a phase II clinical trial on patients in combination with GM-CSF in patients with HER-2-positive breast cancer (NCT00524277). Preliminary results reveal the vaccine is safe and well tolerated, and disease-free survival was improved from 51% with the use of GM-CSF alone compared to 89% with AE37+ GM-CSF [110].

Heat shock protein 70 (HSP70) and a glypican-3 (GPC-3)-derived peptide is undergoing investigation in a phase I clinical trial in solid tumors that express these antigens (UMIN000020440). Preliminary results revealed no severe adverse events, a decrease tumor marker expression in 6 out of 12 patients, and disease control in 5 patients was observed [111].

URLC10-CDCA1-KOC1 multipeptide vaccine uses three HLA-A-24-restricted epitope peptides derived from cancer cells: upregulated lung cancer 10 (URLC10), cell division cycle-associated 1 (CDCA1), and KH domain-containing protein overexpressed in cancer 1 (KOC1). A phase II clinical trial studied this vaccine on patients with esophageal squamous cell carcinoma and found that it is capable of inducing highly specific T cells against these antigens [112].

Other peptide-based vaccines that are undergoing clinical trials but without preliminary results include the mutation-derived tumor antigen (MTA)-based peptide vaccine (NCT02721043); a personalized neoantigen vaccine NEO-PV-01 (NCT02897765); a vaccine composed of GM-CSF and CD40L (GM.CD40l) (NCT02466568); the OCV-C01 composed of peptides derived from KIF20A, VEGFR1, and VEGFR2 (UMIN000007991) [113]; and a Toll-like receptor-2 (TLR2) ligand-synthetic long peptide (SLP) vaccine containing HPV-16 E6 protein long peptides (2014-000658-12) [114].

### In situ vaccines

Poly-ICLC is a synthetic immune danger signal and is specifically a mimic of viral dsRNA that can ligate TLR-3 and trigger cytokine production by DCs with subsequent immune activation and enhancement of the vaccine-induced anti-tumor responses [115]. A phase I/II clinical trial was recently conducted using this TLR ligand as an in situ vaccine in patients with multiple solid tumors including melanoma, breast, and HNSCC. Most of adverse events were mild and limited to the site of application; only 1 out of 8 patients achieved SD for 41 weeks, the remainder of patients showed PD [116].

BO-112 is a synthetic dsRNA administered intratumorally; it activates pro-apoptotic signals MDA-5 and NOXA and increases IFN response genes leading to the anti-tumor activity [117]. Its use is being studied in a phase I clinical trial in patients with palpable malignant tumors including melanoma and breast cancer (NCT02828098). Preliminary results revealed only one episode of reversible thrombocytopenia, with increase in circulating immune cells [117].

### Neoantigen vaccines

Neoantigens are molecules expressed on tumor cell’s surface by DNA mutations that present in tumor cells, but not in normal cells, making it an attractive cancer vaccine target [118]. Although neoantigen cancer vaccines have been long envisioned as ideal, its discovery and evaluation only became feasible recently with the development of highly efficient sequencing. Different from other immunotherapy such as checkpoint inhibitors and CAR T cells, vaccines targeting neoantigens are designed to be individual-specific. This personal vaccine induces a focused T cell response to patient’s specific tumor neoantigens and avoids toxicities caused by damage to normal cells and tissues [119]. Multiple studies are ongoing to further explore this novel exciting approach.

A first-in-human clinical trial on patients with advanced melanoma identified individual mutations and neoantigens and developed a vaccine unique to each patient (IVAC MUTANOME) (NCT02035956). All 13 patients showed T cell response against neoantigens. Eight patients remained recurrence-free for the entire follow-up period (12–23 months). Two of 5 patients that relapsed and achieved objective clinical response, and one of them achieved a CR in multiple metastatic lesions that had been unresponsive to radiation therapy and CTLA-4 blockade. A third patient also achieved CR to vaccination in combination with PD-1 blockade [119]. No major adverse events were reported.

The use of specific neoantigens in personalized vaccines is being explored in a phase I clinical trial in patients with melanoma (NCT01970358). The vaccine targets up to 20 personal tumor neoantigens and preliminary results reveal that 4 of the 6 vaccinated patients had no recurrence of the disease at 25 months after vaccination. The other two patients that experienced recurrence were subsequently managed with anti-PD-1 and achieved a complete tumor regression. Adverse events were mild and consisted of flu-like symptoms, injection site reactions, rash, and fatigue [120].

## Other approaches in immunotherapy

### Targeting myeloid-derived suppressor cells

Myeloid-derived suppressor cells (MDSCs) are immature myeloid cells that promote immunosuppression and favor tumor growth [121]. The TNF-related apoptosis-inducing ligand receptor (TRAIL-R)-2, also known as death receptor (DR)-5, is found on tumor cells and MDSCs, and its activation promotes apoptosis in these populations [122]. The use of the TRAIL-R2 agonist antibody, DS-8273a, is being studied. Results of one trial in patients with solid tumors revealed only mild to moderate adverse events, no dose-limiting toxicities, and a decrease in blood levels of MDSCs [123].

### Cytokine gene therapy

IL-12 has been considered a good option for immunotherapy given its potent anti-tumor effect [124]. This cytokine promotes the activation of NK and T cells and synergizes other cytokines with anti-tumor effects [124].

Ad-RTS-hIL-12 is a replication-incompetent adenovirus engineered to express IL-12. By default, IL-12 expression by this virus is “off,” but with the use of veledimex, gene is activated and lL-12 production is started [125]. The use of Ad-RTS-hIL-12 with veledimex is being studied in patients with advanced gliomas in a phase I clinical trial (NCT02026271). Preliminary results revealed that the most frequent adverse events were mild flu-like symptoms, grade 3 CRS, and grade 3 transaminitis; however, all were reversed upon discontinuation of therapy. A median OS of 12.5 months was also observed [126]. This technology is also being studied in patients with locally advanced or metastatic breast cancer (NCT02423902); however, no preliminary results have been revealed yet.

IL-2 enhances the immune system through the IL-2 receptor (IL-2R) [127]. NKTR-214, an engineered cytokine that specifically stimulates IL-2R, is being investigated on phase I/II clinical trials on solid tumors (NCT02983045, NCT02869295). Preliminary results on the former trial show no dose-limiting toxicities. One patient had a 40% decrease in LDH, and another patient had an unconfirmed CR after only 6 weeks of treatment [128]. The latter trial revealed no dose-limiting toxicities, a tumor size reduction ranging from 10 to 30% in 6 out of 26 patients (23%), and an increase of T cells and NK cells within the tumor microenvironment in 100% of patients [129].

### Targeting tumor microenvironment

Cancer cells require a milieu, known as tumor microenvironment, which allows their growth. This microenvironment consists of immune and nonimmune cells and non-cellular factors that interact among each other and promote a chronic inflammatory, immunosuppressive, and pro-angiogenic ecosystem that favors tumor survival, growth, and dissemination [130]. Some of these factors that have been identified are being investigated as potential therapeutic targets, often in conjunction with other immunotherapy agents.

Indoleamine 2,3-dioxygenase (IDO) is an enzyme that converts tryptophan to kynurenines. These latter promote the formation of Tregs, increase the number of MDSCs, and decrease the activity of CD8 T cells with a resulting inhibitory environment [130, 131]. BMS-986205 is an IDO1 inhibitor that is being studied on a phase I clinical trial in conjunction with a PD-1 inhibitor in patients with advanced solid tumors (NCT02658890). Preliminary results reported mild toxicities except for three cases of grade 3 hepatitis, rash, and hypophosphatemia. No efficacy was described [132]. Indoximod is another IDO inhibitor undergoing phase II clinical trials on melanoma (NCT02073123) and pancreatic (NCT02077881) and castrate-resistant prostate cancer (NCT01560923). Preliminary results reveal an ORR of 52% in patients with melanoma when used with immune checkpoint inhibitors [133]. Patients with pancreatic cancer had an ORR of 37% when indoximod was used with both gemcitabine and nab-paclitaxel [134]. Median PFS increased from 4.1 to 10.3 months in castrate-resistant metastatic prostate cancer compared to placebo [135]. Epacadostat also blocks IDO pathway and is being evaluated on phase I/II clinical trials with multiple solid malignancies (NCT02327078, NCT02178722). Preliminary results have demonstrated an ORR ranging from 75% in melanoma to 4% in CRC. No dose-limiting toxicities were identified [136, 137].

Toll-like receptors (TLRs) are critical in the identification of pathogens but play a complex role in tumorigenesis. TLRs like TLR4 promote cancer progression by promoting inflammation in the microenvironment. TLRs like TLR7/8 and TLR9 promote anti-tumor responses by inducing a “danger signal” and activating the immune system against malignant cells [138]. MEDI9197, a dual agonist of TLR7/8, is under phase I clinical testing in combination with durvalumab and radiation therapy on metastatic or locally advanced solid malignancies (NCT02556463). Preliminary results show that the agent is overall safe with only mild adverse events. No efficacy data has been yet reported [139]. PG545 (pixatimod, pINN) is an agonist of TLR9/IL-12 tested in a phase I clinical trial in patients with advanced solid tumors [140]. Results show that 3 out of 23 patients developed dose-limiting toxicities and the best response achieved was a 24-week SD and a DCR of 38%. Polyinosinic-polycytidylic acid polylysine carboxymethylcellulose (poly-ICLC) is a potent TLR3 agonist that was studied in combination with radiation in a phase I clinical trial in patients with hepatocellular carcinoma not eligible for surgery [141]. Intratumoral injection was found to be safe with mostly grade I–II adverse events, a PFS of 66% at 6 months and 28% at 24 months and an OS after 1 year was 69% and 38% after 2 years [141].

Arginine is an amino acid required for T cell activation and proliferation. Malignant cells produce high levels of arginase and deplete arginine interfering with immune activation [142]. CB-1158, an arginase inhibitor, is being studied in a phase I clinical trial alone and in combination with a PD-1 inhibitor in patients with advanced solid tumors (NCT02903914). Preliminary results reveal no dose-limiting toxicities, > 90% of arginase inhibition, and up to a 4-fold increase in plasma arginine levels [143].

### Oncolytic peptides

LTX-315 is a cytotoxic peptide that damages the tumor-mitochondrial membranes and triggers caspase-independent necrosis leading to a massive release of tumor antigens and to an increase in TIL activity [144]. A phase I clinical trial is investigating this agent as monotherapy or in combination with ICIs in patients with metastatic solid tumors, particularly melanoma and breast cancer (NCT01986426). Preliminary results showed that 2 patients achieved a CR, 5 patients had a decrease of > 50% of the tumor size, and 8 patients achieved SD [145].

## Conclusions

Cancer immunotherapy has changed the landscape of modern oncology in varied cancer types. Immunotherapy with checkpoint inhibitors has significantly improved the clinical outcomes in some, but not all patients. This is likely due to individual differences in immunogenicity of tumor and immunosuppressive tumor microenvironments. The understanding of emerging novel immunotherapeutic approaches beyond immune checkpoints discussed above will likely open the opportunities to patients with cancers that have failed to respond to an immune checkpoint inhibitor alone. Furthermore, combination therapies targeting different immune mechanisms will likely to better modulate the immune systems to boost an anti-tumor response.

The development of tumor-directed antibodies, antibody-drug conjugates, CAR T cells, oncolytic viruses, vaccines, and even genetic therapy has allowed for a more targeted and tumor-specific therapy rather than a non-specific cytolytic chemotherapy or radiation therapy. Next wave of clinical trials are already evaluating the combinations of immunotherapy agents from different classes. Immune-related side effects, cost of treatment, lack of response biomarkers, and tumor relapse are remaining challenges. Nevertheless, this rapidly advancing field is becoming the most promising treatment component of current oncologic therapy.

## Abbreviations

AARC: American Association of Cancer Research
ACT: Adoptive cell transfer
ADC: Antibody drug conjugate
ALL: Acute lymphoid leukemia
AML: Acute myeloid leukemia
ASCO: American Society of Clinical Oncology
B-ALL: B cell acute lymphoblastic leukemia
BCMA: B cell maturation antigen
BiTEs: Bispecific T cell engaging antibodies
CAR: Chimeric antigen receptor
CEA: Carcinoembryonic antigen
CEACAM: CEA cell adhesion molecule
CLL: Chronic lymphocytic leukemia
CR: Complete response
CRC: Colorectal
CTLA-4: Cytotoxic T lymphocyte-associated molecule-4
DART: Dual affinity re-targeting
DC: Dendritic cells
DCR: Disease control rate
GCC: Guanylyl cyclase C
GPC-3: Glypican-3
gpNMB: Glycoprotein non-metastatic b
HCC: Hepatocellular carcinoma
HNSCC: Head and neck squamous cell carcinoma
HSP70: Heat shock protein 70
LAG-3: Lymphocyte activation gene 3
MDS: Myelodysplastic syndrome
MDSCs: Myeloid-derived suppressor cells
MILs: Marrow-infiltrating lymphocytes
MM: Multiple myeloma
MTAs: Mutation-derived tumor antigens
MVA: Modified Vaccinia Ankara
NHL: Non-Hodgkin’s lymphoma
NK: Natural killer
NKG2D: Natural killer group 2D
NSCLC: Non-small cell lung carcinoma
OR: Objective response
ORR: Objective response rate
OS: Overall survival
PD-1: Programmed cell death receptor-1
PD-L1: Programmed cell death ligand-1
PFS: Progression-free survival
PR: Partial response
PSMA: Prostate-specific membrane antigen
SCLC: Small cell lung cancer
SD: Stable disease
TBI: Total body irradiation
TCR: T cell receptor
TILs: Tumor-infiltrating lymphocytes
TIM-3: T cell immunoglobulin
TLPLDC: Tumor lysate, particle-loaded, dendritic cell
TLR: Toll-like receptor
URLC10: Upregulated lung cancer 10
VEGFR-2: Vascular endothelial growth factor receptor-2
VISTA: V-domain Ig suppressor of T cell activation
WT-1: Wilms tumor gene-1
